# Consistent scaling of whole-shoot respiration between Moso bamboo (*Phyllostachys pubescens*) and trees

**DOI:** 10.1007/s10265-021-01320-5

**Published:** 2021-06-11

**Authors:** Mofei Wang, Shigeta Mori, Yoko Kurosawa, Juan Pedro Ferrio, Keiko Yamaji, Kohei Koyama

**Affiliations:** 1 The United Graduate School of Agricultural Science, Iwate University, Morioka, Iwate 020-8550 Japan; 2 Faculty of Agriculture, Yamagata University, Tsuruoka, Yamagata 997-8555 Japan; 3 Aragon Agency for Research and Development (ARAID), 50018 Zaragoza, Spain; 4 Department of Forest Resources, Agrifood Research and Technology Centre of Aragon (CITA), 50059 Zaragoza, Spain; 5 Graduate School of Life and Environmental Sciences, University of Tsukuba, Tsukuba, Ibaraki 305-8577 Japan; 6 Department of Agro-environmental Science, Obihiro University of Agriculture and Veterinary Medicine, Obihiro, Hokkaido 080-8555 Japan

**Keywords:** Division of labor among bamboo shoots, Metabolic scaling, Organ respiration, *Phyllostachys pubescens*, Whole-shoot respiration

## Abstract

Both Moso bamboo (*Phyllostachys pubescens*) and tree forests have a large biomass; they are considered to play an important role in ecosystem carbon budgets. The scaling relationship between individual whole-shoot (i.e., aboveground parts) respiration and whole-shoot mass provides a clue for comparing the carbon budgets of Moso bamboo and tree forests. However, nobody has empirically demonstrated whether there is a difference between these forest types in the whole-shoot scaling relationship. We developed whole-shoot chambers and measured the shoot respiration of 58 individual mature bamboo shoots from the smallest to the largest in a Moso bamboo forest, and then compared them with that of 254 tree shoots previously measured. For 30 bamboo shoots, we measured the respiration rate of leaves, branches, and culms. We found that the scaling exponent of whole-shoot respiration of bamboo fitted by a simple power function on a log–log scale was 0.843 (95 % CI 0.797–0.885), which was consistent with that of trees, 0.826 (95 % CI 0.799–0.851), but higher than 3/4, the value typifying the Kleiber’s rule. The respiration rates of leaves, branches, and culms at the whole-shoot level were proportional to their mass, revealing a constant mean mass-specific respiration of 1.19, 0.224, and 0.0978 µmol CO_2_ kg^− 1^ s^− 1^, respectively. These constant values suggest common traits of organs among physiologically integrated ramets within a genet. Additionally, the larger the shoots, the smaller the allocation of organ mass to the metabolically active leaves, and the larger the allocation to the metabolically inactive culms. Therefore, these shifts in shoot-mass partitioning to leaves and culms caused a negative metabolic scaling of Moso bamboo shoots. The observed convergent metabolic scaling of Moso bamboo and trees may facilitate comparisons of the ecosystem carbon budgets of Moso bamboo and tree forests.

**Supplementary Information:**

The online version contains supplementary material available at 10.1007/s10265-021-01320-5.

## Introduction

Plant size is one of the most important factors that explain the relationship between carbon supply and demand at the whole-plant to ecosystem scales (Collalti et al. 2019; O’Leary et al. 2019). The respiration rate of terrestrial plants scales with plant body mass, and the scaling relationship is generally modelled by a simple power function on log–log coordinates. The value of 3/4, which has been generally accepted as the scaling exponent of the function, was originally suggested by Kleiber (1932) and theoretically modelled by West et al. (1997). However, the use of scaling exponents remains controversial (Banavar et al. 2014; Cheng et al. 2010; Glazier 2005, 2018; Yagi et al. 2010). One of the reasons for this controversy may be the limited number of studies based on reliable measurements of respiration of individual trees, because it is difficult to measure the respiration of a large tree. To reach a certain conclusion on the controversy, it is necessary to accumulate reliable measurements of respiration of individuals ranging from tiny seedlings to large trees. For various organisms including animals and plants, the scaling exponents are between 0.75 and 1 from the embryo to mature stages (Makarieva et al. 2008; Mori et al. 2010; O’Leary et al. 2019; Peng et al. 2010; Reich et al. 2006). Reich et al. (2006) suggested isometric scaling by measurements of the respiration rates from seedlings to young trees. They also suggested that the scaling relationship between individual respiration rates and their mass was similar within or along species and that they were not affected by growth conditions. However, Peng et al. (2010) reported that the metabolic scaling of shrubs was affected by growth rate. Mori et al. (2010) demonstrated that the scaling exponent varies from 1 in small plants to 3/4 in larger trees based on the measurements of whole-shoot (aboveground parts) respiration from tropical to boreal forests; similar results were reported by Cheng et al. (2010). Thus, whether the scaling exponent of 3/4 can be applied across phylogenies and environments is still empirically and theoretically debated including for trees and bamboo (Banavar et al. 2014; Poorter et al. 2015).

Unlike trees, bamboo does not have secondary cambium and annual rings. Therefore, although both trees and bamboo grow to a large size, they are expected to differ substantially in individual physiological traits that control growth. However, the differences in the respiration rate between bamboo and trees have not been empirically evaluated (Yuen et al. 2017; Zhou et al. 2011). Bamboo is a woody grass and is currently classified under the tribe Bambuseae, subfamily Bambusoideae within the family Poaceae (Isagi et al. 2016). There are 1250–1500 bamboo species within 75–107 genera in the world (Scurlock et al. 2000; Yuen et al. 2017). They cover approximately 31.5 million ha of land, accounting for 0.8 % of the world’s total forested area (Song et al. 2011; Yuen et al. 2017). Among various bamboo species, Moso bamboo (*Phyllostachys pubescens* (Carrière) J.Houz.) covers the largest area, 3.37 million ha, accounting for 70 % of China’s bamboo-growing area (Song et al. 2011; Yuen et al. 2017; Isagi et al. 2016) analyzed the genetic diversity of Moso bamboo for the entire distribution range from Japan to China using microsatellite markers and found that the samples from Japan and China comprise an identical clone. They reported that the clone was distributed over more than 2800 km and the estimated biomass was approximately 6.6 × 10^11^ kg (Isagi et al. 2016). As Moso bamboo has expanded its distribution in various places and invaded adjacent forests, it is now considered a threat to biodiversity (Takano et al. 2017).

Several comparative studies on structure and function such as respiratory consumption, structural development, carbon dynamics, and carbon sequestration have been conducted with the aim to elucidate the differences between Moso bamboo and trees mainly at the ecosystem level (Isagi et al. 1997; Isagi and Torii 1998; Isagi et al. 2016; Mao et al. 2016; Song et al. 2016). However, to the best of our knowledge, empirical studies on the respiration rate of whole-bamboo shoot have not been performed (Isagi et al. 1997), because the whole-bamboo shoots are too large to enclose in a measurement chamber. Considering that any bamboo and tree forest communities are composed of small to large shoots, it is necessary to clarify scaling of individual shoot respiration. Furthermore, a bamboo forest is composed of clonally integrated different sized shoots, that is, ramets. Among ramets within clonal communities such as bamboo forests, they can generally exchange various resources including carbon, thus, realizing higher production under heterogenous environmental conditions (Liu et al. 2016; Saitoh et al. 2002; Stuefer et al. 1996; Tomimatsu et al. 2020). Unlike the integrated bamboo shoots, independent trees have been considered to compete for capturing light energy. We predicted that these differences between bamboo and trees would cause additional differences in the scaling of respiration as the energy use of individual shoots and each organ changes in proportion with size of the individual.

The scaling of respiration of individual shoots and each organ will provide fundamental clues for comparing carbon dynamics and the development of shoots between bamboos and trees (Collalti et al. 2019; O’Leary et al. 2019; Salomón et al. 2020). To clarify the differences in scaling, we measured the respiration rate of 58 individual Moso bamboo shoots and their total organs. We compared them with the shoot respiration rate of 254 trees composed of 67 species (Mori et al. 2010), which were previously measured using the same methods employed in this study.

## Materials and methods

**Fig. 1:**
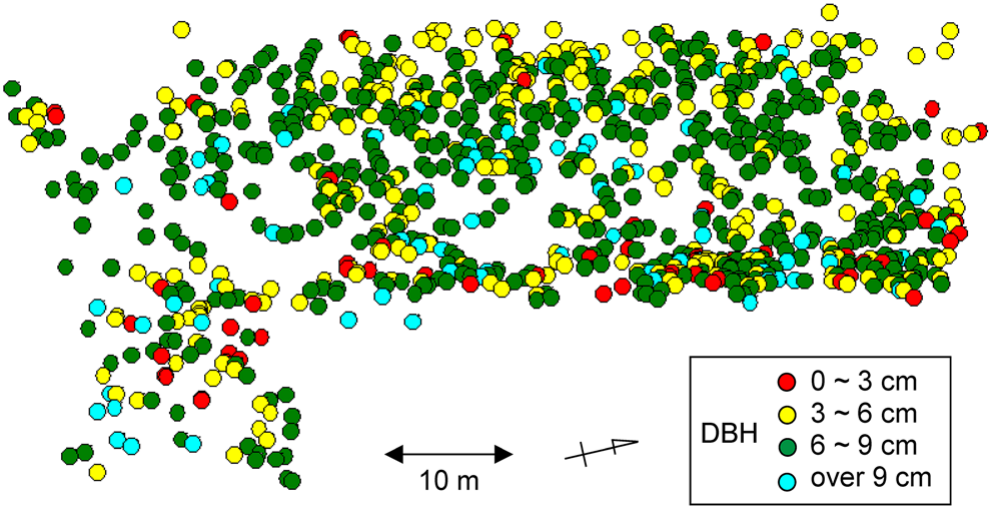
Location map of all standing bamboo shoots classified by DBH (diameter at 1.3 m above ground) in 2018, including bamboo forest edge. The bamboo forest is surrounded by tree forests in the south and grasslands in the north. The western region is under a slightly dark condition shaded by evergreen trees, and the eastern region has a parking lot that does not shade the bamboo forest

This study was conducted in a Moso bamboo forest at the Faculty of Agriculture, Yamagata University in Tsuruoka, Yamagata, Japan (38° 73ʹ N, 138° 82ʹ E). The average temperature and annual precipitation in the AMeDAS observation station of the Japan Meteorological Agency in Tsuruoka (ca. 0.6 km from the study site) from 1981 to 2010 were 12.9 °C and 2097.5 mm, respectively. The research site is located at an altitude of 16 m above sea level and has a total area of approximately 792 m^2^. The stand density of bamboo was 6415 shoots ha^− 1^ in 2015. The mean shoot height and diameter at breast height (DBH) of the bamboo forest were 9.68 (SD = 2.66) m and 6.77 (SD = 1.92) cm, respectively. The location map of all shoots, including the ones grown at the edge of the Moso bamboo forest is shown in Fig. 1.

We selected 58 bamboo shoots of various sizes spanning from the smallest to the largest throughout the forest (from the edge to the centre). The fresh mass of bamboo shoots ranged from 0.275 to 31.0 kg; their age ranged from 1 (1 year after emergence) to 5 + years (5 years or over 5 years) as shown in Table S1. We did not measure the respiration rate of current-year shoots. All the 58 shoots were used for whole-shoot respiration measurements, and 30 out of the 58 shoots were selected to measure the respiration rate of total leaves, branches, and culms (Table S1). To compare the respiration rate of Moso bamboo shoots with that of trees, we used our data of whole-shoot respiration rate of trees comprising 67 species (*n* = 254, Mori et al. 2010), collected using the same methods that we followed previously (Kurosawa et al. 2021; Mori et al. 2010).

We measured the respiration rate of bamboo shoots after the growing season, from late July to mid**-**September in 2017 and 2018. Respiration was measured using custom-made chambers developed by Mori et al. (2010). Immediately after felling bamboo shoots, plants were sprayed with water and covered with black sheets to prevent transpiration. Each measurement was taken within about 20 min after felling the shoots. First, we removed all branches from the shoots and measured the respiration rate. In this step, the leaves were kept attached to the branches to avoid leaf desiccation. Next, the leaves were completely removed from the branches, and the respiration rate of total branches was measured. Finally, we measured the total culm respiration rate.

**Fig. 2:**
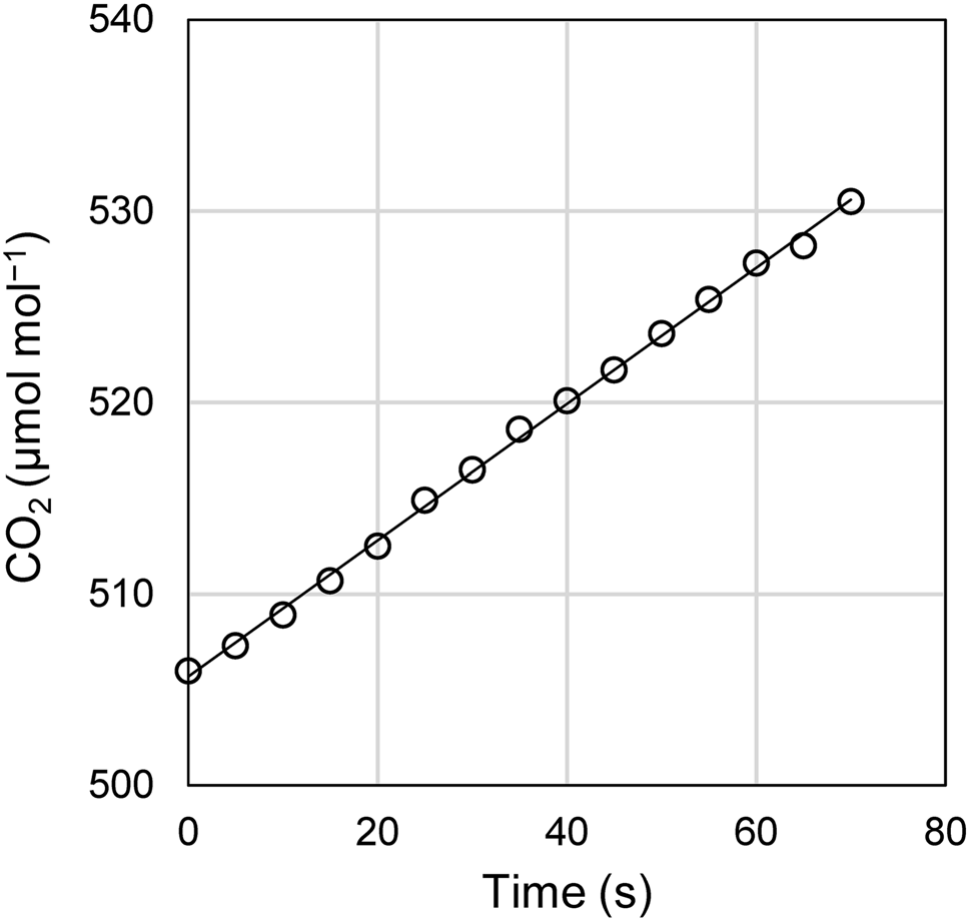
Example of CO_2_ increments d*C*/d*t* (µmol mol s^− 1^) as measured within the chamber was fitted by the equation *C* = 0.3372 *t* + 505.7, *r*^2^ = 0.99

**Fig. 3:**
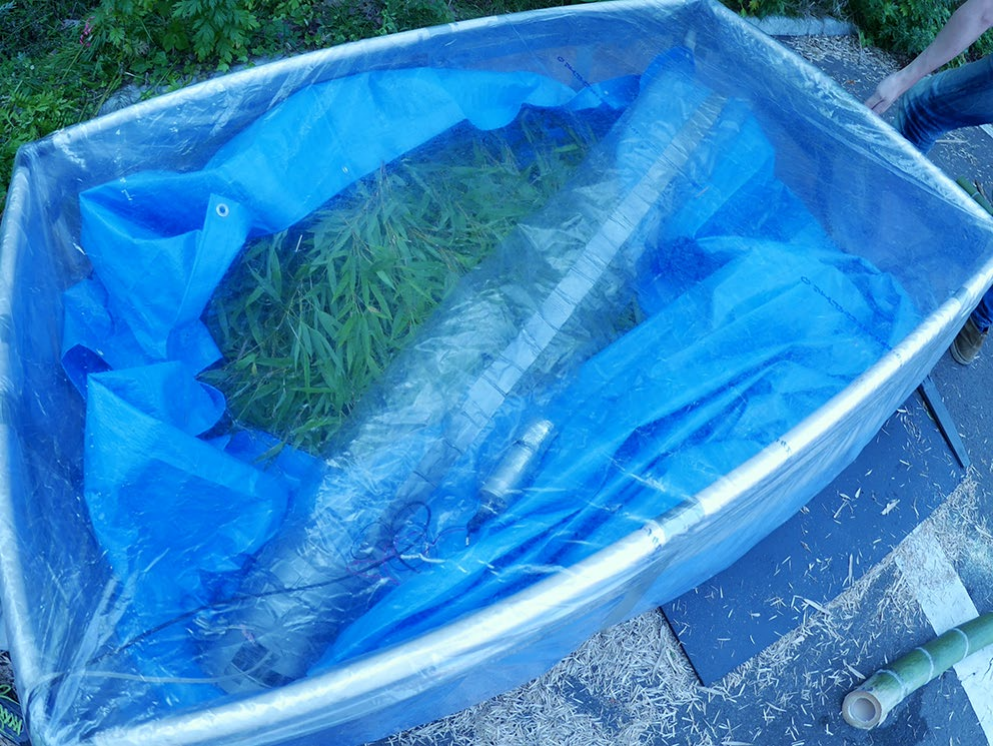
Photograph showing the closed air circulation chamber (0.96 m^3^) used to measure respiration of a bamboo branch. Inside the chamber is the sample, a DC fan with a duct, and a CO_2_ sensor. After sealing, the chamber was maintained dark by covering with a black sheet

The increase rate of CO_2_ concentration within the chamber was measured every 5 s for 30–300 s with an infrared CO_2_ analyzer (GMP343; Vaisala, Helsinki, Finland) (Fig. 2). The temperature variation inside the chambers during the measurements was at the most 1 °C. The respiration rates were adjusted to that at a temperature of 20 °C with the assumption that Q_10_ = 2 to evaluate the potential of shoot respiration during summer even if there is a seasonal variation in Q_10_ (Glazier 2020; Hoque et al. 2010). For accurate measurements, we prepared chambers of various volumes (0.004–0.96 m^3^), and changed the chambers depending on the size of materials. A fan with a duct and a CO_2_ sensor were placed inside each chamber (Fig. 3). We confirmed that the separation of plant parts did not affect whole-shoot respiration rates, as reported in previous studies (Dang et al. 1997; Mitchell et al. 1999; Mori et al. 2010; Reich et al. 1998).

### Data analysis

The relationship between respiration rate *R* (µmol CO_2_ s^− 1^) of an individual organism and its body mass *M* (kg) is generally modelled by the following simple power function:

1 $$R = {aM}^b$$

where *a* (µmol CO_2_ kg^− 1^ s^− 1^) is the intercept or *R* at 1 kg *M*, and *b* (dimensionless) is the scaling exponent or the slope on the log–log coordinates (Kleiber 1932; West et al. 1997). Equation (1) generally represents the metabolic rate as a function of body mass under various constraints. From a metabolic perspective, using fresh mass as a proxy for body size is important because all the active components such as enzymes are contained in the liquid phase and are the ultimate source of metabolic activity (Huang et al. 2019; Kurosawa et al. 2021; Makarieva et al. 2008; Thakur et al. 2018). In fact, metabolic scaling is usually based on fresh mass for various phylogenies in studies on evolutionary biology and metabolic ecology (Ferrio et al. 2018; Makarieva et al. 2008; Sibly et al. 2012).

Size scaling of respiration rates was done using a simple power function with log–log coordinates, based on reduced major axis (RMA) regression (Niklas and Hammond 2014) of the log-transformed version of Eq. (1), using PAST software (Hammer et al. 2001). Statistical significances of scaling components were determined using 95 % confidence intervals (CIs). That is, groups significantly differed if there was no overlap among the 95 % CIs. We also performed ordinary least squares (OLS) regression for all scaling relationships to compare the results by RMA regression. The use of OLS regression did not change our results.

## Results

### Size scaling of whole-shoot respiration vs. whole-shoot fresh mass in bamboo and trees

**Fig. 4:**
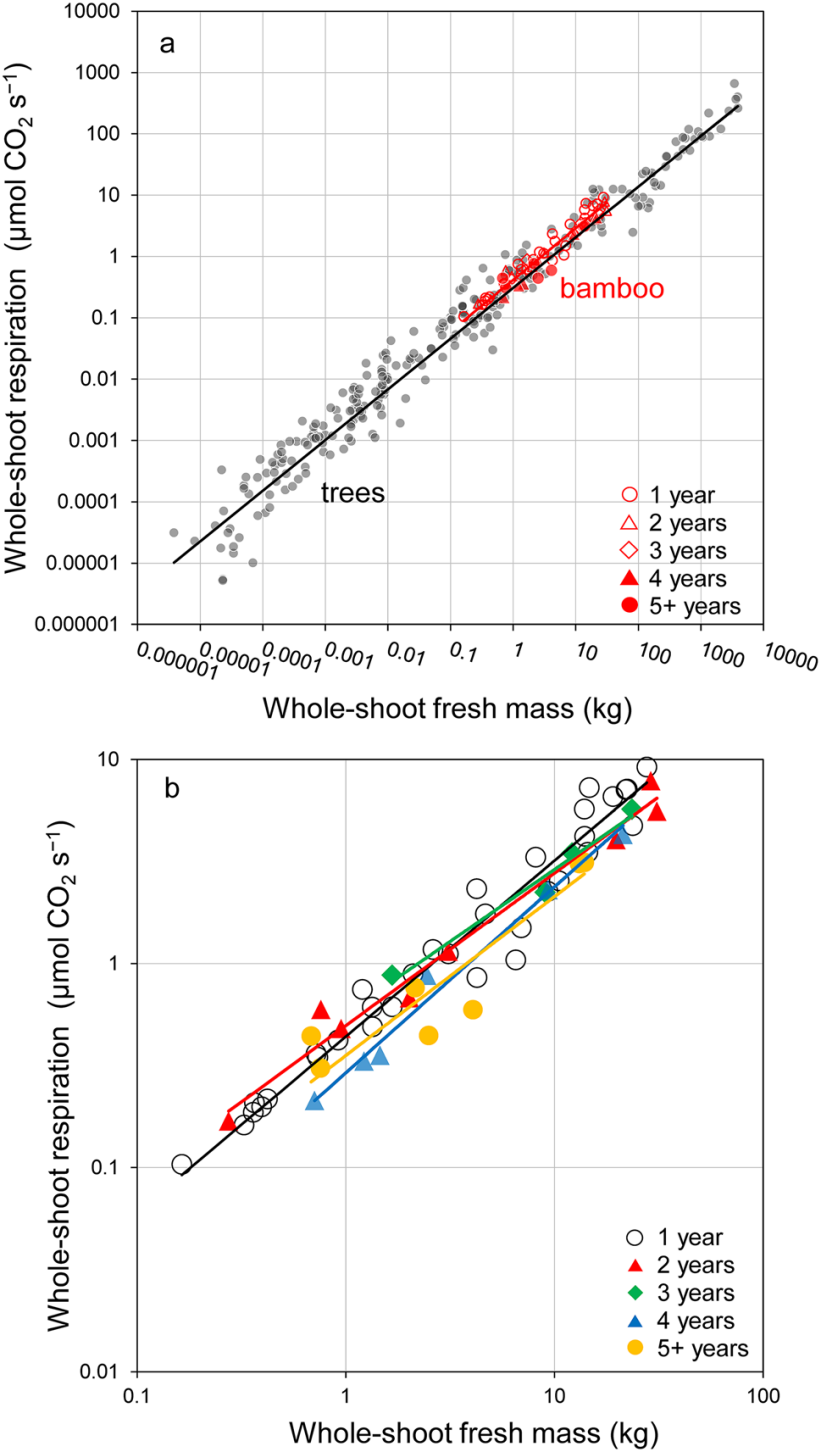
**a** Results of the reduced major axis (RMA) analysis showing the relationships between whole-shoot respiration and whole-shoot fresh mass of bamboo and trees (Mori et al. 2010). Details are compiled in Table 1. **b** Results of the reduced major axis (RMA) analysis showing the relationships between whole-shoot respiration and whole-shoot fresh mass of bamboo of different ages. Details are compiled in Supplementary Table S2

We determined the relationship between whole-shoot respiration and whole-shoot mass in bamboo and trees using log–log coordinates (Fig. 4a; Table 1). For bamboo, there were no apparent differences in the respiration rate among the age groups (Fig. 4b, Table S2); therefore, we used the same equation for all age groups. Bamboo and trees had consistent negative scaling exponents (i.e., *b* < 1) for whole-shoot respiration rates (Table 1). The relationship between bamboo whole-shoot respiration rate and fresh mass was similar to that of trees. For both bamboo and trees, the *b* values were significantly higher than the value predicted by West et al. (1997) (*b* = 0.75). Therefore, the smallest bamboo shoots had the highest mass-specific respiration on the regression line (Fig. 4), which was approximately 2.3 times larger than that of the largest bamboo shoot.

**Table 1:** Results of the reduced major axis (RMA) analysis for scaling of whole-shoot respiration rate (µmol CO_2_ s^− 1^) and fresh mass (kg) of bamboo and trees (Mori et al. 2010) on log–log coordinates, with *p* < 0.001

	Number of observations	Exponent *b*	95 % CI of *b*	Intercept *a* (CO_2_ kg^− 1^ s^− 1^)	95 % CI of *a* (CO_2_ kg^− 1^ s^− 1^)	*r* ^2^
Bamboo shoots^a^	58	0.843	0.797–0.885	0.414	0.379–0.459	0.941
Tree shoots^b^	254	0.826	0.799–0.851	0.306	0.267–0.348	0.980

^a^ Use of the ordinary least squares (OLS) regression for bamboo shoots provided similar results (exponent *b* = 0.818, 95 % CI = 0.774–0.861; Intercept *a* = 0.426, 95 % CI = 0.394–0.467, *r*^2^ = 0.941, *n* = 58, *p* < 0.001) with the results of the RMA analysis

^b^ Mori et al. (2010)

### **Size scaling of respiration of leaves, branches, and culms vs. organ fresh mass in bamboo**

**Fig. 5:**
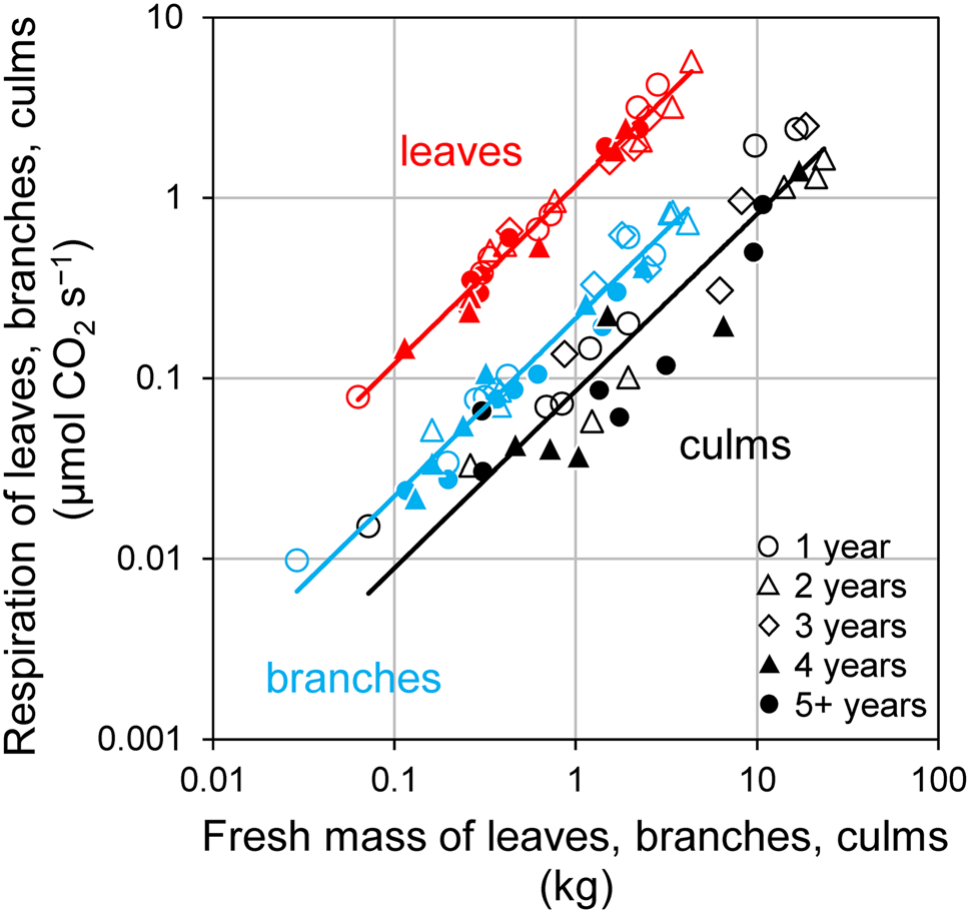
Results of the reduced major axis (RMA) analysis showing the relationships among respiration and fresh mass of bamboo leaves, branches, and culms. In each analysis, *n* = 30. Details are compiled in Table 2A

To further examine bamboo shoot respiration, the scaling of respiration rates of individual organs was determined. The respiration rate of leaves, branches, and culms increased almost proportionally with mass (Fig. 5; Table 2A). The scaling exponent *b* consistently had a value of almost 1. Thus, mass-specific respiration rates of bamboo leaves, branches, and culms were constant regardless of their organ mass, with the mean values of 1.19 (SD = 0.0736), 0.224 (SD = 0.0221), and 0.0978 (SD = 0.0205) µmol CO_2_ kg^− 1^ s^− 1^, respectively. This means that the mass-specific respiration of leaves was 12.2 times higher than that of culms.

**Table 2:** Results of the reduced major axis (RMA) analysis on log–log coordinates for scaling of (A) respiration rate (µmol CO_2_ s^− 1^) in relation to fresh mass (kg) of leaves, branches, and culms; (B) fresh mass of leaves, branches, and culms (kg) in relation to whole-shoot fresh mass (kg); and (C) respiration rate (µmol CO_2_ s^− 1^) of leaves, branches, and culms in relation to whole-shoot fresh mass (kg). For all regression analysis, *n* = 30 and *p* < 0.001

	Exponent *b*	95 % CI of *b*	Intercept *a* (CO_2_ kg^− 1^ s^− 1^)	95 % CI of *a* (CO_2_ kg^− 1^ s^− 1^)	*r* ^2^
(A) Organ respiration vs. organ fresh mass					
Leaves	0.990	0.932–1.047	1.172	1.095–1.252	0.976
Branches	0.983	0.887–1.059	0.215	0.193–0.237	0.957
Culms	0.981	0.840–1.093	0.085	0.068–0.111	0.875
(B) Organ fresh mass vs. whole-shoot fresh mass					
Leaves	0.787	0.714–0.851	0.246	0.211–0.294	0.926
Branches	0.894	0.849–0.948	0.174	0.155–0.193	0.978
Culms	1.122	1.081–1.158	0.533	0.489–0.586	0.993
(C) Organ respiration vs. whole-shoot fresh mass					
Leaves	0.780	0.691–0.862	0.292	0.240–0.363	0.898
Branches	0.881	0.788–0.962	0.039	0.035–0.045	0.934
Culms	1.100	0.941–1.225	0.046	0.036–0.063	0.894

### Organ fresh mass and respiration rate vs. whole-shoot fresh mass in bamboo

**Fig. 6:**
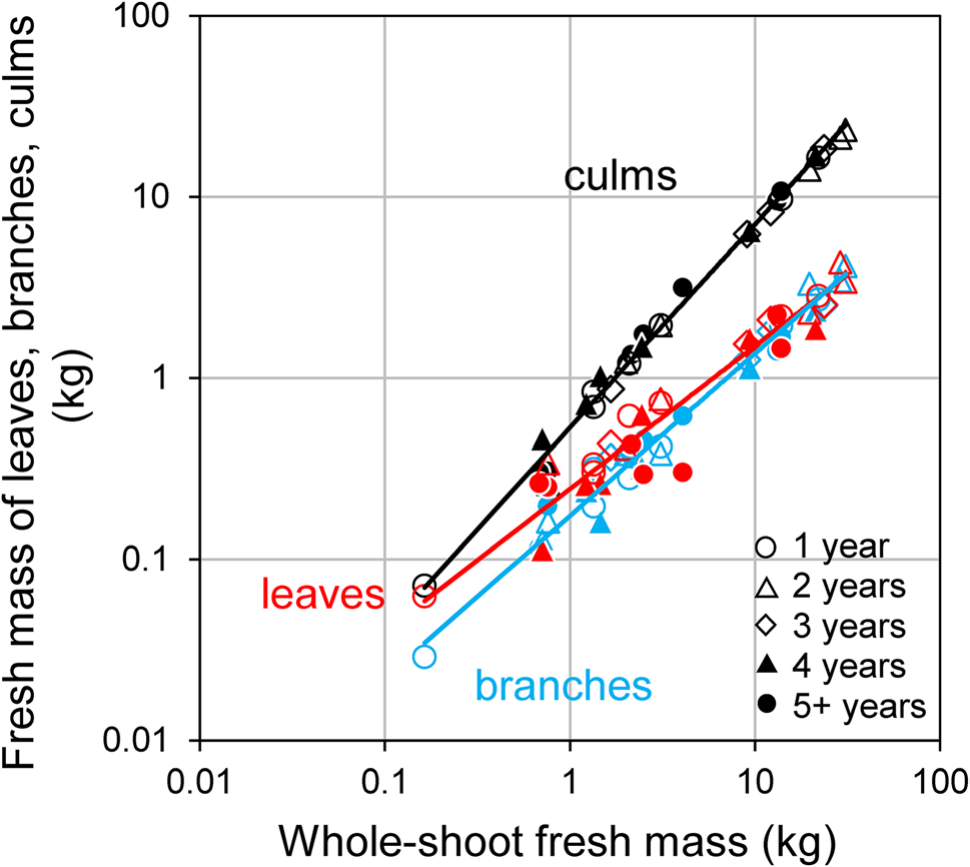
Results of the reduced major axis (RMA) analysis showing the relationships among the fresh mass of bamboo leaves, branches, culms and whole-shoot fresh mass. In each analysis, *n* = 30. Details are compiled in Table 2B

Among the three organs, the fresh mass of total culms was always the highest across the entire range of shoot fresh mass (Fig. 6). As the scaling exponent of culm fresh mass vs. whole-shoot fresh mass was significantly positive (i.e., *b* > 1) at the 95% CI level as compiled in Table 2B, the larger the whole-shoot fresh mass, the larger the fresh mass partitioning of culms in shoot fresh mass. The fraction of culm fresh mass increased from 42.7 to 77.2 % on the regression line. On the contrary, both scaling exponents of leaves and branches vs. whole-shoot fresh mass were allometrically negative (i.e., *b* < 1). On the regression line, as shoot fresh mass increased, the leaf mass fraction decreased from 36.0 to 11.3 % and the branch mass fraction decreased from 21.3 to 11.5%.

**Fig. 7:**
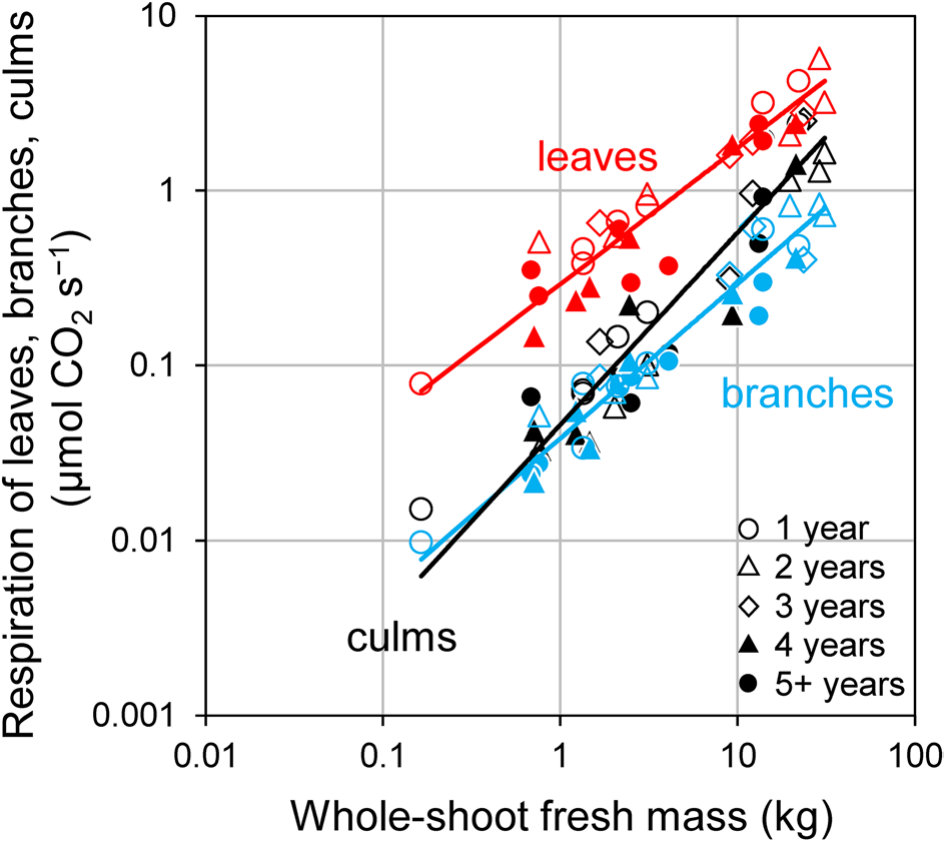
Results of the reduced major axis (RMA) analysis showing the relationships among respiration of bamboo leaves, branches, culms and whole-shoot fresh mass. In each analysis, *n* = 30. Details are compiled in Table 2C

The respiration rate in the total leaves was always the highest across the entire range of whole-shoot fresh mass (Fig. 7). The scaling exponents of respiration in the leaves and branches vs. whole-shoot fresh mass were significantly negative (i.e., *b* < 1) as compiled in Table 2C. The larger the whole-shoot fresh mass, the smaller the fraction of leaf respiration in shoot respiration. On the regression line, the fraction of leaf respiration decreased from 83.5 to 60.2 %. The fraction of branch respiration in shoot respiration was relatively constant, and it ranged from 9.4 to 11.4 %. On the contrary, the exponent of culm respiration vs. whole-shoot fresh mass was not significant but somewhat positive (i.e., *b* > 1), and therefore the fraction of culm respiration in shoot respiration increased from 7.1 to 28.4 % with increasing of whole-shoot fresh mass on the regression line.

## Discussion

Moso bamboo differs from trees in not only growth traits but also phylogeny. Despite these differences, we found that Moso bamboo and trees were similar in the scaling of whole-shoot respiration (Fig. 4a; Table 1). Whether the general metabolic scaling can be applied across phylogenies and environments is still being debated (Banavar et al. 2014; Makarieva et al. 2008; Poorter et al. 2015; Reich et al. 2006). The present study results support the general scaling of whole-shoot respiration vs. whole-shoot biomass with similar slopes among various terrestrial plants including bamboo and trees.

In the present study, we empirically demonstrated that for both bamboo and trees the scaling exponents of whole-shoot respiration vs. whole-shoot fresh mass were statistically slightly greater than 3/4. Similarly, slopes higher than 3/4 have been reported in other studies (Cheng et al. 2010; Glazier 2005; Peng et al. 2010; Reich et al. 2006). To elucidate the reason for the equivalency of the scaling exponents between bamboo and trees, it is necessary to compare scaling of respiration and mass among organs in bamboo shoots (Enquist et al. 2007). Several studies in various organisms have suggested that the negative allometry (i.e., scaling exponent < 1) of the metabolic rate is partially due to the increase in the relative masses of organs with low metabolic rates as in the stems and roots (Atkin 2010; Cheng et al. 2010; Glazier 2014; Kurosawa et al. 2021; Mori et al. 2010; Oikawa and Itazawa 2003). One of the reasons for this size-dependent shift (i.e., the larger the fresh mass of shoots, the lower the mass-specific respiration rate) in trees may be the physico-chemical constraints (Atkin 2010; Ballesteros et al. 2018; Kurosawa et al. 2021; Mori et al. 2010), mainly gravity, which becomes increasingly important as plants grow (Enquist et al. 2007; Enquist and Bentley 2012). As trees have secondary cambium, the larger trees accumulate more tissue with a low respiratory activity in non-photosynthetic organs such as trunks, roots, and branches (Cheng et al. 2010; Mori and Hagihara 1988, 1995; West et al. 1999). Similar to trees, the larger bamboo shoots, the larger allocation of shoot mass to inactive culms, and the smaller to active leaves (Fig. 6; Table 2B). However, only in bamboo, we found a size-independent (i.e., constant) mass-specific respiration rate within each organ (Fig. 5; Table 2A). Thus, the shift in mass-specific respiration in each organ among various sized shoots is clearly different between bamboo and trees. The difference is probably because bamboo forests are composed of physiologically integrated ramets, whereas tree forests are composed of independent individuals.

Several studies have shown that clonal plant communities have carbon translocation among ramets, which are physiologically integrated, and attain high production under heterogeneous environments (Liu et al. 2016; Saitoh et al. 2002; Stuefer et al. 1996; Tomimatsu et al. 2020). Recently, Song et al. (2016) reported a 28-fold seasonal variation in the non-structural carbonhydrate (NSC) concentration in the culms of a Moso bamboo forest and suggested that culms have an important role as a significant storage organ of NSCs for rapid growth of newly recruited shoots in the following year (Song et al. 2016). Thus, we suggested that the constant mass-specific respiration rate of leaves, branches, and culms of Moso bamboo might be caused by the active translocation of NSCs.

The larger the Moso bamboo shoots, the larger the partitioning of mass and respiration for culms, whereas the smaller the bamboo shoots, the larger the partitioning for leaves as shown in Figs. 6 and 7. In previous studies on herbaceous clonal plant communities, when the ramets were under high light condition and connected to a shaded counterpart, the biomass partitioning for leaves was larger (Roiloa et al. 2007; Stuefer et al. 1996). Several studies on the management of Moso bamboo forests have reported that small bamboo shoots with a large number of leaves tended to grow in the mowed area accompanied with high levels of light (Ishii 2009; Iwasawa and Hirose 2015; Torii 2018). Actually, we observed that the smaller bamboo shoots tended to occur near forest edges, as shown in Fig. 1. The smaller shoots might have a role to fill gaps of Moso bamboo crown, similar to the adventitious branches within a tree. Therefore, Moso bamboo forest might realize a type of division of labor, that is, the smaller shoots relatively focused on carbon acquisition and the larger shoots on storage, due to the active translocation of carbon among various sized ramets.

Moso bamboo is considered to have the greatest potential in fixing CO_2_ in Asia, but it has been difficult to determine whether bamboo forests are carbon sinks or carbon sources without a study of carbon budgets in bamboo forest ecosystems (Lin et al. 2017; Wen et al. 2011; Zhou et al. 2005, 2011). However, the study of carbon budget of bamboo forests has been limited, including measurements of respiration of bamboo at not only the whole-shoot level but also the total-organ level (Chen et al. 2018; Isagi et al. 1997). In the present study, we demonstrated for the first time that scaling of whole-shoot respiration vs. fresh shoot mass is significantly consistent with that of trees. In future studies, the consistent negative scaling of bamboo and tree shoots may provide a new comparative understanding of the differences in the carbon budget between them.

## Electronic Supplementary Material

Below is the link to the electronic supplementary material.

Supplementary Material 1
