# Supplementary material for: Consistent scaling of whole-shoot respiration between Moso bamboo (Phyllostachys pubescens) and trees
Source: J Plant Res. 2021 Jun 11;134(5):989–97. doi: 10.1007/s10265-021-01320-5 (PMC8364903; doi:10.1007/s10265-021-01320-5)
Supplement: Supplementary file 1 — Supplementary Material 1 [file 10265_2021_1320_MOESM1_ESM.docx]

Supplementary Information

**Consistent scaling of whole-shoot respiration between Moso bamboo (*Phyllostachys pubescens*) and trees**

Mofei Wang^1, 2^, Shigeta Mori^2, 1^, Yoko Kurosawa^1, 2^, Juan Pedro Ferrio^3, 4^, Keiko Yamaji^5^, Kohei Koyama^6^

^1^ The United Graduate School of Agricultural Science, Iwate University, Morioka, Iwate 020-8550, Japan

^2^ Faculty of Agriculture, Yamagata University, Tsuruoka, Yamagata 997-8555, Japan

^3^ Aragon Agency for Research and Development (ARAID), Zaragoza 50018, Spain

^4^ Department of Forest Resources, Agrifood Research and Technology Centre of Aragon (CITA), Zaragoza 50059, Spain

^5^ Graduate School of Life and Environmental Sciences, University of Tsukuba, Tsukuba, Ibaraki 305-8577, Japan

^6^ Department of Agro-environmental Science, Obihiro University of Agriculture and Veterinary Medicine, Obihiro, Hokkaido 080-8555, Japan

**Corresponding Author**

Shigeta Mori

E-mail: [morishigeta@tds1.tr.yamagata-u.ac.jp](mailto:morishigeta@tds1.tr.yamagata-u.ac.jp)

Table S1 Compilation of measured data of 58 bamboo shoots

| Fresh mass (kg) | Height (cm) | DBH (cm)^a^ | Age (years) |  |
| --- | --- | --- | --- | --- |
| 0.164 | 225 | 1.0 | 1 | W^b^ |
| 0.275 | 284 | 2.3 | 2 |  |
| 0.325 | 282 | 1.4 | 1 |  |
| 0.361 | 295 | 1.5 | 1 |  |
| 0.365 | 306 | 1.5 | 1 |  |
| 0.396 | 301 | 1.5 | 1 |  |
| 0.422 | 366 | 1.5 | 1 |  |
| 0.683 | 397 | 2.5 | 5+ | W^b^ |
| 0.710 | 342 | 2.5 | 4 | W^b^ |
| 0.720 | 310 | 1.8 | 1 |  |
| 0.735 | 335 | 1.8 | 1 |  |
| 0.757 | 439 | 2.5 | 5+ | W^b^ |
| 0.762 | 257 | 1.7 | 2 | W^b^ |
| 0.918 | 473 | 2.4 | 1 |  |
| 0.953 | 370 | 2.0 | 2 |  |
| 1.202 | 417 | 2.9 | 1 |  |
| 1.225 | 425 | 2.7 | 4 | W^b^ |
| 1.341 | 572 | 2.2 | 1 | W^b^ |
| 1.342 | 440 | 2.4 | 1 | W^b^ |
| 1.460 | 429 | 3.5 | 4 | W^b^ |
| 1.669 | 465 | 3.8 | 1 |  |
| 1.670 | 424 | 3.2 | 3 | W^b^ |
| 2.021 | 520 | 3.9 | 2 | W^b^ |
| 2.100 | 551 | 3.9 | 1 | W^b^ |
| 2.150 | 433 | 3.7 | 5+ | W^b^ |
| 2.450 | 475 | 3.9 | 4 | W^b^ |
| 2.500 | 577 | 3.9 | 5+ | W^b^ |
| 2.621 | 489 | 4.1 | 1 |  |
| 3.100 | 480 | 4.6 | 1 | W^b^ |
| 3.100 | 535 | 4.6 | 2 | W^b^ |
| 4.070 | 880 | 5.8 | 5+ | W^b^ |
| 4.239 | 698 | 4.5 | 1 |  |
| 4.250 | 757 | 4.6 | 1 |  |
| 4.675 | 660 | 4.8 | 1 |  |
| 6.523 | 831 | 5.5 | 1 |  |
| 6.926 | 866 | 5.7 | 1 |  |
| 8.150 | 970 | 6.4 | 1 |  |
| 9.050 | 885 | 7.1 | 3 | W^b^ |
| 9.300 | 905 | 7.2 | 1 |  |
| 9.350 | 998 | 8.4 | 4 | W^b^ |
| 10.550 | 1060 | 6.8 | 1 |  |
| 12.150 | 840 | 8.0 | 3 | W^b^ |
| 13.200 | 1059 | 8.4 | 5+ | W^b^ |
| 13.900 | 1105 | 7.7 | 5+ | W^b^ |
| 13.906 | 896.2 | 8.5 | 1 | W^b^ |
| 13.970 | 1105 | 7.5 | 1 |  |
| 14.480 | 1131 | 7.5 | 1 |  |
| 14.704 | 920 | 8.7 | 1 |  |
| 19.100 | 1036 | 9.3 | 1 |  |
| 19.750 | 1019 | 10.0 | 2 | W^b^ |
| 21.350 | 1110 | 9.9 | 4 | W^b^ |
| 22.057 | 1217 | 9.8 | 1 | W^b^ |
| 22.300 | 1310 | 9.0 | 1 |  |
| 23.600 | 1264 | 9.2 | 3 | W^b^ |
| 23.700 | 1230 | 10.4 | 1 |  |
| 27.760 | 1166 | 12.3 | 1 |  |
| 29.000 | 1169 | 11.2 | 2 | W^b^ |
| 30.986 | 1201 | 9.5 | 2 | W^b^ |

^a^ DBH: diameter at breast height (measured at 1.3 m height above ground)

^b^ W: For 30 shoots, after measuring organ respiration, we used the sum of organ respiration for individual shoot respiration

Table S2 Results of the reduced major axis (RMA) analysis for scaling of whole-shoot respiration rate (µmol CO_2_ s^−1^) and fresh mass (kg) of bamboo of different ages on log–log coordinates, with *p* < 0.001

| Age | Number of observations | Exponent *b* | 95 % CI of *b* | Normalization constant *a* | 95 % CI of *a* | *r*^2^ |
| --- | --- | --- | --- | --- | --- | --- |
| 1 year | 33 | 0.863 | 0.819–0.907 | 0.438 | 0.410–0.476 | 0.958 |
| 2 years | 8 | 0.747 | 0.667–0.849 | 0.497 | 0.396–0.592 | 0.976 |
| 3 years | 4 | 0.706 | 0.492–0.858 | 0.569 | 0.412–0.756 | 0.975 |
| 4 years | 6 | 0.912 | 0.607–1.065 | 0.291 | 0.239–0.335 | 0.984 |
| 5+ years | 7 | 0.779 | 0.459–1.247 | 0.354 | 0.253–0.738 | 0.837 |
